# Supplementary material for: Unpaid caregiving and mental health during the COVID-19 pandemic—A systematic review of the quantitative literature
Source: PLoS One. 2024 Apr 18;19(4):e0297097. doi: 10.1371/journal.pone.0297097 (PMC11025839; doi:10.1371/journal.pone.0297097)
Supplement: S1 File — (DOCX) [file pone.0297097.s002.docx]

**Unpaid Caregiving and Mental Health during the COVID-19 Pandemic - a Systematic Review of the quantitative literature**

# Supplement 2 –Search Strategy (3 items)

#### TIERED SEARCH STRATEGY

#### DETAILED SEARCH STRATEGY

#### MINOR PROTOCOL AMENDMENT

## Tiered search strategy

| Tier 1 | Mental health (Outcome) | mental health/ or depression/ or anxiety/ or ((Mental and health) or depress* or anxiety or "psychological stress" or "psychological distress" or psychological).mp |
| --- | --- | --- |
| Tier 2 | Unpaid care (Exposure) | (“Informal unpaid car*” or “informal car*” or "unpaid car*" or “family car*” or carer or caregiv* or “unpaid childcar*”).mp |
| Tier 3 | COVID-19 | disease outbreaks/ or epidemics/ or pandemics/ or (coronavirus* or 2019-ncov or ncov19 or ncov-19 or 2019-novel Cov or ncov or covid or covid19 or covid-19 or covid 2019 or "coronavirus 2" or sars-cov2 or sars-cov-2 or sarscov2 or sarscov-2 or sars-coronavirus2 or sars-coronavirus-2 or SARS-like coronavirus* or coronavirus-19 or corona virus* or novel coronavirus*).mp. |

## Detailed search strategy

**Ovid MEDLINE(R) and Epub Ahead of Print, In-Process, In-Data-Review & Other Non-Indexed Citations**

1 (mental and health).mp.

2 (depress* or anxiety).mp.

3 ("psychological stress" or "psychological distress" or psychological).mp.

4 mental health/

5 depression/

6 anxiety/

7 1 or 2 or 3 or 4 or 5 or 6

8 ("informal unpaid car*" or "informal car*" or "unpaid car*" or "family car*" or carer or caregiv* or "unpaid childcar*").mp.

9 disease outbreaks/ or epidemics/ or pandemics/ or (coronavirus* or 2019-ncov or ncov19 or ncov-19 or 2019-novel Cov or ncov or covid or covid19 or covid-19 or covid 2019 or "coronavirus 2" or sars-cov2 or sars-cov-2 or sarscov2 or sarscov-2 or sars-coronavirus2 or sars-coronavirus-2 or SARS-like coronavirus* or coronavirus-19 or corona virus* or novel coronavirus*).mp.

10 7 and 8

11 9 and 10

12 limit 11 to yr="2020 -Current"

**APA PsycInfo**

1 (mental and health).mp.

2 (depress* or anxiety).mp.

3 ("psychological stress" or "psychological distress" or psychological).mp.

4 mental health/

5 depression/

6 anxiety/

7 1 or 2 or 3 or 4 or 5 or 6

8 ("informal unpaid car*" or "informal car*" or "unpaid car*" or "family car*" or carer or caregiv* or "unpaid childcar*").mp.

9 disease outbreaks/ or epidemics/ or pandemics/ or (coronavirus* or 2019-ncov or ncov19 or ncov-19 or 2019-novel Cov or ncov or covid or covid19 or covid-19 or covid 2019 or "coronavirus 2" or sars-cov2 or sars-cov-2 or sarscov2 or sarscov-2 or sars-coronavirus2 or sars-coronavirus-2 or SARS-like coronavirus* or coronavirus-19 or corona virus* or novel coronavirus*).mp.

10 7 and 8

11 9 and 10

12 limit 11 to yr="2020 -Current"

**Embase Classic+Embase**

1 (mental and health).mp.

2 (depress* or anxiety).mp.

3 ("psychological stress" or "psychological distress" or psychological).mp.

4 mental health/

5 depression/

6 anxiety/

7 1 or 2 or 3 or 4 or 5 or 6

8 ("informal unpaid car*" or "informal car*" or "unpaid car*" or "family car*" or carer or caregiv* or "unpaid childcar*").mp.

9 disease outbreaks/ or epidemics/ or pandemics/ or (coronavirus* or 2019-ncov or ncov19 or ncov-19 or 2019-novel Cov or ncov or covid or covid19 or covid-19 or covid 2019 or "coronavirus 2" or sars-cov2 or sars-cov-2 or sarscov2 or sarscov-2 or sars-coronavirus2 or sars-coronavirus-2 or SARS-like coronavirus* or coronavirus-19 or corona virus* or novel coronavirus*).mp.

10 7 and 8

11 9 and 10

12 limit 11 to yr="2020 -Current"

**SCOPUS**

( ( TITLE-ABS-KEY ( "mental health" OR depress* OR anxiety OR "psychological stress" OR "psychological distress" OR psychological ) ) W/10 ( TITLE-ABS-KEY ( "informal unpaid car*" OR "informal car*" OR "unpaid car*" OR "family car*" OR carer OR caregiv* OR "unpaid childcar*" ) ) ) AND ( TITLE-ABS-KEY ( coronavirus* OR 2019-ncov OR ncov19 OR ncov-19 OR "2019-novel Cov" OR ncov OR covid OR covid19 OR covid-19 OR "covid 2019" OR "coronavirus 2" OR sars-cov2 OR sars-cov-2 OR sarscov2 OR sarscov-2 OR sars-coronavirus2 OR sars-coronavirus-2 OR sars-like AND coronavirus* OR coronavirus-19 OR corona AND virus* OR novel AND coronavirus* OR pandemic ) ) AND PUBYEAR > 2019

**Web of Science**

((TS=("mental health" OR depress* OR anxiety OR "psychological stress" OR "psychological distress" OR psychological)) AND TS =(("informal unpaid car*" OR "informal car*" OR "unpaid car*" OR "family car*" OR carer OR caregiv* OR "unpaid childcar*")) AND TS=(( coronavirus* OR 2019-ncov OR ncov19 OR ncov-19 OR "2019-novel Cov" OR ncov OR covid OR covid19 OR covid-19 OR "covid 2019" OR "coronavirus 2" OR sars-cov2 OR sars-cov-2 OR sarscov2 OR sarscov-2 OR sars-coronavirus2 OR sars-coronavirus-2 OR sars-like AND coronavirus* OR coronavirus-19 OR corona AND virus* OR novel AND coronavirus* OR pandemic) )) *| Timespan: 2020-01-01 to 2023-12-31 (Publication Date*

## Minor Protocol Amendment

During the search/study selection stage of the review, we (the authors) discussed, agreed upon and implemented a minor amendment to our comparator/control inclusion criteria. Where the comparator(s)/control stipulated in original protocol was "no (or lower levels of) informal unpaid care", it was amended to "no informal unpaid care" (removing "lower levels"). This was decided to reduce heterogeneity between the included studies and ensure a truly unexposed comparator for our review. The Prospero register was amended accordingly.
