# Supplementary material for: Unpaid caregiving and mental health during the COVID-19 pandemic—A systematic review of the quantitative literature
Source: PLoS One. 2024 Apr 18;19(4):e0297097. doi: 10.1371/journal.pone.0297097 (PMC11025839; doi:10.1371/journal.pone.0297097)
Supplement: S2 File — (DOCX) [file pone.0297097.s003.docx]

**Unpaid Caregiving and Mental Health during the COVID-19 Pandemic - a Systematic Review of the quantitative literature**

# Supplement 3 – Quality Assessment (3 items)

#### NEWCASTLE-OTTAWA QUALITY ASSESSMENT SCALE (AMENDED)

#### PRE-DEFINED CONSIDERATIONS FOR QUALITY ASSESSMENT PROCESS

#### NEWCASTLE-OTTAWA QUALITY ASSESSMENT SCORES (ACROSS DOMAINS)

## Newcastle-Ottawa Quality Assessment Scale (amended)

**Selection**

1) Representativeness of the exposed cohort

a) truly representative of the average unpaid carer **🟑**

b) somewhat representative of the average unpaid carer **🟑**

c) selected group of users e.g., nurses, volunteers

d) no description of the derivation of the cohort

2) Selection of the non-exposed cohort

a) drawn from the same community as the exposed cohort **🟑**

b) drawn from a different source

c) no description of the derivation of the non-exposed cohort

3) Ascertainment of exposure

a) secure record (e.g., surgical records) **🟑**

b) structured interview or structured questionnaire **🟑**

c) written self-report

d) no description

4) Non-respondents

a) Comparability between respondents and non-respondents’ characteristics is established, **or** the response rate is satisfactory (equal or over 50%)^1^ **🟑**

b) The response rate is unsatisfactory, **and** the comparability between respondents and non-respondents is unsatisfactory

c) No description of the response rate or the characteristics of the respondents and non-respondents

**Comparability**

1) Comparability of cohorts based on the design or analysis

a) study controls for minimal set of confounding factors (age, gender, SEP, employment) **🟑**

b) study meets additional comparability considerations: For example, authors conducted a robust statistical test (such as IPTW/fixed effects) maximising exchangeability and/or controlled for important additional confounding factors such as ethnicity, household structure, long-term health condition, other COVID/pandemic specific considerations **🟑**

**Outcome**

1) Assessment of outcome

a) Independent assessment or confirmation of outcome via medical records; or clinical interview **🟑**

b) Ascertained via reliable, validated instruments e.g., standardised scale such as General Health Questionnaire **🟑**

c) Self-reported information without use of validated instrument

d) No description

2) Was follow-up long enough for outcomes to occur (as per Lacey et al 2022)^2^

a) yes - Longitudinal **🟑**

b) no – Cross-sectional

3) Adequacy of follow up

a) complete follow up - all subjects accounted for **🟑**

b) subjects lost to follow up unlikely to introduce bias (small number lost/ > 75% follow up, or description provided of those lost) **🟑**

c) follow up rate < 75% and no description of those lost

d) Cross-sectional – no follow-up

e) no statement

4) Statistical test:

a) The statistical test used to analyse the data is clearly described and appropriate, and the measurement of the association is presented, including confidence intervals and/or the probability level (p value) **🟑**

b) The statistical test is not appropriate, not described, or incomplete

**Overall quality rating:** 10 points*

A study can be awarded a maximum of one star for each numbered item within the Selection and Outcome categories. A maximum of two stars can be given for Comparability.

Low RoB (Higher quality) NOS score ≥ 8/10

Some RoB (Medium quality) NOS score 5-7/10

High RoB (Low quality) NOS score of ≤ 4/10

** The maximum number of stars a study with longitudinal design could score was ten, whereas the maximum score for a cross-sectional study design was 8/10 stars.*

## Pre-defined considerations for quality assessment process

| **General considerations for all included studies** | |
| --- | --- |
| Research context: | Evaluate the association between informal unpaid caregiving and mental health during the COVID-19 pandemic |
| Participants | No restriction on population age (young carers included as well)  No restrictions on geographical setting or country level socioeconomic development are imposed. This review will be limited to population-based research. |
| Exposure | Informal unpaid caring (for individuals who are either temporarily or permanently ill or physically and/or mentally disabled, and the elderly and children (before they are legal adults)). |
| Control/comparison | No informal unpaid care/Non carers |
| Minimal set of confounders | - Gender - Age - SEP or any level of disadvantage (education, and/or occupational level and/or income) - Employment status and/or Paid work hrs*     If <18 - young carers - then it will be parental income and employment as 3^rd^ and 4^th^ confounding factors  *Controlling for employment as part of the minimal set is only expected in working-age populations. Surveys restricted to older populations (60yrs+) are considered to have controlled for employment through sampling. |
| Additional comparability considerations | - Additional important confounders: Household structure (for young carers would at least consider whether they live in a household with 1 or 2 parents, as this could determine both the caring load and their mental health); Ethnicity/migrant status; Family members with disability (illness); Other COVID/pandemic related confounders. - Methodology/study design that enhances exchangeability. |
| Criteria for ascertaining accuracy of exposure measurement | "Unpaid caregiving/care work is variously defined. For the purposes of this review and following the precedent of Friedemann-Sanchez & Griffin (2011),^3^ we consider informal caregiving or unpaid care work to be “the provision of unpaid personal services to meet the physical, mental, and emotional needs that allow a dependent person to function at a socially determined acceptable level of capability, comfort, and safety. We consider dependants to be all children (before they are legal adults), all those either temporarily or permanently ill or physically and/or mentally disabled, and the elderly”.  This exposure will be mainly self-reported by participants in panel survey data - assess whether well-defined or not. May be categorical or continuous variables. |
| Included outcomes | Validated mental health measures; depression, anxiety, psychological distress/wellbeing. |
| Factors to consider when evaluating outcome assessment | Self-reported MH outcomes may be unreliable. Assess whether it is a validated measure (e.g., SF-36/MHI-5 or K6). May be categorical or numerical.  Informal unpaid caring status will be measured at any point during pandemic, with mental health outcomes measured at or after the exposure to unpaid or informal caregiving. Consider when exposure and outcome are measured, i.e. – is study cross-sectional or longitudinal design (and what the follow up time is). |

##

## Newcastle-Ottawa Quality Assessment Scores (across domains)

| **Author YEAR** | **Representativeness of the exposed cohort** | **Selection of the non-exposed cohort** | **Ascertainment of exposure** | **Non-response** | **Comparability of cohorts (design and/or analysis)** | **Assessment of outcome** | **Was follow-up long enough for outcomes to occur** | **Adequacy of follow up of cohorts** | **Statistical test** | **Total** |
| --- | --- | --- | --- | --- | --- | --- | --- | --- | --- | --- |
| Allen 2022 | 1 | 1 | 1 | 1 | 2 | 1 | 0 | 0 | 1 | **8** |
| Amerio 2021 | 0 | 1 | 1 | 0 | 0 | 1 | 0 | 0 | 1 | **4** |
| Beach 2021 | 1 | 1 | 1 | 0 | 2 | 1 | 0 | 0 | 1 | **7** |
| Costi 2023 | 1 | 1 | 1 | 0 | 2 | 1 | 1 | 1 | 1 | **9** |
| DiGessa 2022 | 1 | 1 | 1 | 1 | 2 | 1 | 1 | 1 | 1 | **10** |
| Fusar-Poli 2022 | 1 | 1 | 1 | 0 | 0 | 1 | 0 | 0 | 1 | **5** |
| Ganadjian 2022 | 1 | 1 | 1 | 0 | 1 | 1 | 0 | 0 | 1 | **6** |
| Hammerberg 2020 | 1 | 1 | 1 | 1 | 1 | 1 | 0 | 0 | 0 | **6** |
| Hung 2021 | 0 | 1 | 1 | 0 | 0 | 1 | 0 | 0 | 1 | **4** |
| Landi 2022 | 1 | 1 | 1 | 1 | 0 | 1 | 0 | 0 | 1 | 6 |
| Liu 2021 | 1 | 1 | 1 | 0 | 2 | 1 | 0 | 0 | 1 | **7** |
| Mak 2022 | 1 | 1 | 1 | 0 | 2 | 1 | 1 | 0 | 0 | **7** |
| McGarrigle 2022 | 1 | 1 | 1 | 1 | 1 | 1 | 1 | 1 | 0 | **8** |
| Nakanishi 2022 | 1 | 1 | 1 | 0 | 0 | 1 | 1 | 1 | 1 | **7** |
| Newby 2020 | 0 | 1 | 1 | 0 | 1 | 1 | 0 | 0 | 1 | **5** |
| Noguchi 2021 | 1 | 1 | 1 | 0 | 1 | 1 | 0 | 0 | 1 | **6** |
| Park 2021 | 1 | 0 | 1 | 1 | 2 | 1 | 1 | 0 | 1 | **8** |
| Rodrigues 2021 | 1 | 1 | 1 | 0 | 1 | 1 | 0 | 0 | 1 | **6** |
| Russell 2022 | 0 | 1 | 1 | 0 | 0 | 1 | 0 | 0 | 0 | **3** |
| Whitley 2021 | 1 | 1 | 1 | 0 | 0 | 1 | 1 | 0 | 1 | **6** |
| Wilson 2022 | 1 | 1 | 1 | 0 | 0 | 1 | 0 | 0 | 1 | **5** |
| Wister 2022 | 1 | 1 | 1 | 1 | 1 | 1 | 1 | 0 | 0 | **7** |
| Yoshioka 2021 | 1 | 1 | 1 | 0 | 1 | 1 | 0 | 0 | 1 | **6** |

# References

1. Krieger N, LeBlanc M, Waterman PD, Reisner SL, Testa C, Chen JT. Decreasing Survey Response Rates in the Time of COVID-19: Implications for Analyses of Population Health and Health Inequities. *American Journal of Public Health* 2023: e1-e4.

2. Lacey RE, Xue B, McMunn A. The mental and physical health of young carers: a systematic review. *Lancet Public Health* 2022; **7**(9): e787-e96.

3. Friedemann-Sánchez G, Griffin JM. Defining the Boundaries between Unpaid Labor and Unpaid Caregiving: Review of the Social and Health Sciences Literature. *Journal of Human Development and Capabilities* 2011; **12**(4): 511-34.
