# Supplementary material for: Unpaid caregiving and mental health during the COVID-19 pandemic—A systematic review of the quantitative literature
Source: PLoS One. 2024 Apr 18;19(4):e0297097. doi: 10.1371/journal.pone.0297097 (PMC11025839; doi:10.1371/journal.pone.0297097)
Supplement: S3 File — (DOCX) [file pone.0297097.s004.docx]

**Unpaid Caregiving and Mental Health during the COVID-19 Pandemic - a Systematic Review of the quantitative literature**

# Supplementary 4 - Data synthesis

## Albatross plot explanation

When meta-analysis is not possible, the albatross plot (utilising precise p-value, effect direction (SMD) and total sample size) plots the study sample size against p-values, with results separated according to the observed direction of the effect, allowing p-values to be interpreted in the context of the study sample size.^1^ Small studies appear towards the bottom of the plot and larger studies towards the top. Effect contours are superimposed providing an indication of the magnitude of effect for individual studies as well as for the association as a whole.^1^ Broadly, if studies mostly have a similar effect size, then points will fall around a single contour line. Whereas, if there is substantial heterogeneity of effect size, then the points are scattered across contours. Importantly, albatross plots are not designed to estimate the magnitude of an association precisely, they merely provide a visual aid in interpreting the results where a meta-analysis is not feasible.^1^

Specific to our plots, exposure groups were subdivided by caregiving type (according to the studies included in the review) into adult/general caregiving (Fig 2a) and childcare (Fig 2c), with an albatross plot for each pertaining to the depression/psychological distress outcome. We note that for the Caregiving and Depression Albatross plot (Fig 2a), four of the calculated SMD’s were calculated from unadjusted estimates of the association between caring and mental health,^2-5^ whilst ten were adjusted for confounders. Additionally, sufficient adult caregiving studies examined anxiety as an outcome to also construct an albatross plot for this subgroup (Fig 2b). Given most studies did not stratify their results by gender, we had insufficient data to meaningfully stratify our albatross plots by gender, however we did include estimates for both men and women (colour coded) where available in the overall albatross plots. We also note that in studies where more than one estimate was reported, we chose the effect estimate for levels of exposure most comparable with the other included studies.

## Fisher’s meta-analysis of combining p values explanation.

Fisher’s meta-analysis of combining p-values was also employed as part of the data synthesis. Combining p-values addresses the question “is there evidence that there is an effect in at least one study?”^6^ Fisher’s method utilises one-sided p-values as these contain information about the direction of effect.^6^ In addition to computing an overall Fisher’s p for all studies in the review (p<0.001), there were sufficient studies for the studies for subgroups adult caregiving and depression (Fig 2a) and adult caregiving and anxiety (Fig 2b) to compute subgroup Fisher’s p, both of which were p<0.001. All these results suggest strong evidence of unpaid caregiving being associated with worse MH outcomes.

#

# References

1. Harrison S, Jones HE, Martin RM, Lewis SJ, Higgins JPT. The albatross plot: A novel graphical tool for presenting results of diversely reported studies in a systematic review. *Res Synth Methods* 2017; **8**(3): 281-9.

2. Allen J, Uekusa S, Alpass FM. Longitudinal Cohort Study of Depression and Anxiety Among Older Informal Caregivers Following the Initial COVID-19 Pandemic Response in Aotearoa New Zealand. *Journal of aging and health* 2022; **34**(4-5): 653-65.

3. Beach SR, Schulz R, Donovan H, Rosland AM. Family Caregiving During the COVID-19 Pandemic. *Gerontologist* 2021; **61**(5): 650-60.

4. Fusar-Poli L, Surace T, Meo V, et al. Psychological well-being and family distress of Italian caregivers during the COVID-19 outbreak. *Journal of community psychology* 2022; **50**(5): 2243-59.

5. Wilson E, Onwumere J, Hirsch C. Psychological Processes Associated With Resilience in UK-Based Unpaid Caregivers During the COVID-19 Pandemic. *Clinical psychology in Europe* 2022; **4**(4): e10313.

6. McKenzie JE, Brennan SE. Synthesizing and presenting findings using other methods. In: Higgins JPT TJ, Chandler J, Cumpston M, Li T, Page MJ, Welch VA (editors). editor. Cochrane Handbook for Systematic Reviews of Interventions version 63: Cochrane; 2022.
