# Supplementary material for: Unpaid caregiving and mental health during the COVID-19 pandemic—A systematic review of the quantitative literature
Source: PLoS One. 2024 Apr 18;19(4):e0297097. doi: 10.1371/journal.pone.0297097 (PMC11025839; doi:10.1371/journal.pone.0297097)
Supplement: S1 Table — (DOCX) [file pone.0297097.s005.docx]

**Unpaid Caregiving and Mental Health during the COVID-19 Pandemic - a Systematic Review of the quantitative literature**

# Supplement 5 – Full Text Exclusions

**Table. Articles excluded after full-text review with reasons for exclusion.**

|  | **Title** | **Reason** |
| --- | --- | --- |
| 1 | Parmar, J., & Anderson, S. (2021). THE IMPACTS OF THE COVID-19 PANDEMIC ON FAMILY CAREGIVERS' CARE WORK, ANXIETY, AND LONELINESS. Innovation in aging, 5, 550-550. | No full text |
| 2 | Cona, M.S., Dalu, D., Rulli, E., Galli, F., Rota, S., Ferrario, S., et al. (2021). COVID-19 and cancer: How outpatients (pts) and their caregivers (CGs) face to the pandemic-A survey from the midst of the Italian outbreak. Journal of Clinical Oncology, 39. | No full text |
| 3 | Rasiah, S., Cohen-Silver, J., Lebovic, G., Maguire, J., & Freeman, S. (2022). Mental Health of Children and Caregivers Who Use School-Based Health Centers During the COVID-19 Pandemic in Toronto, Canada. JOURNAL OF DEVELOPMENTAL AND BEHAVIORAL PEDIATRICS, 43, E144-E144. | No full text |
| 4 | Turkkan, O.N., Yildiz, N., Cicek, N., Bodur, E.D., Guven, S., Sak, M., et al. (2021). ANXIETY, DEPRESSION AND COPING OF CHILDREN WITH CHRONIC KIDNEY DISEASE AND THEIR CAREGIVERS DURING THE COVID-19 PANDEMIC. PEDIATRIC NEPHROLOGY, 36, 3337-3337. | No full text |
| 5 | Turpin, K.V.L., Foland, E., Patton, T., Guterl, J., Zapakin, M., & Greenamyer, J. (2022). Impact of COVID-19 on cancer caregivers: Results from the Global Carer Well-being Index. Journal of Clinical Oncology, 40. | No full text |
| 6 | Verkleij, M., Noij, L., Hashimoto, S., Terheggen-Lagro, S., Altenbug, J., & Haarman, E. (2021). Mental health during COVID-19 in Dutch adolescents and adults with cystic fibrosis (CF) or primary ciliary dyskinesia (PCD) and their caregivers. EUROPEAN RESPIRATORY JOURNAL, 58. | No full text |
| 7 | Figueiredo, D., Silva, D., Sousa, H., Bartolo, A., & Ribeiro, O. (2022). POS-896 QUALITY OF LIFE OF FAMILY CAREGIVERS OF PATIENTS UNDERGOING IN-CENTER HEMODIALYSIS DURING THE COVID-19 PANDEMIC: PRELIMINARY RESULTS. Kidney International Reports, 7, S388. | No full text |
| 8 | Asfuroglu, P., Eyuboglu, T.S., Aslan, A.T., Gursoy, T.R., Senkalfa, B.P., Soysal, S., et al. (2022). Change in the anxiety levels of children with cystic fibrosis and their mothers at the beginning of the COVID-19 pandemic and after 1 year. Journal of Cystic Fibrosis, 21, S138-S139. | No full text |
| 9 | Blanc, J., Seixas, A., Turner, A., Chung, A., Jean-Louis, G., & Coffey, B. (2021). 50.9 Caregiver Status Predicts PTSD Among New Yorkers During the US COVID-19 Pandemic. Journal of the American Academy of Child and Adolescent Psychiatry, 60, S252. | No full text |
| 10 | Castro-Tejada, G., Diaz-Garelli, F., Mauricio, A.H.C., Rodriguez-Lockward, A.L., Hernandez, I., Dominguez, J., et al. (2020). Burden and anxiety in caregivers of Alzheimeras disease patients during the COVID-19 pandemic: A cross-sectional study. European Geriatric Medicine, 11, S16. | No full text |
| 11 | De Young, A., Alisic, E., Cobham, V., Donovan, C., Hoehn, E., March, S., et al. (2022). COVID-19 UNMASKED: LONGITUDINAL COHORT STUDY EXAMINING MENTAL HEALTH OF YOUNG CHILDREN AND THEIR CAREGIVERS DURING THE PANDEMIC. Australian and New Zealand Journal of Psychiatry, 56, 132-133. | No full text |
| 12 | Dobi, F., Kabili, E., Malile, E., Dragoti, F., Dorre, I., & Jolla, A. (2021). Evaluation of depression symptoms among caregivers of children that take therapy in the national center for children rehabilitation and treatment during COVID-19 pandemic. EUROPEAN PSYCHIATRY, 64, S287-S287. | No full text |
| 13 | Leleszi-Trobert, A.M., Bagyura, M., & Szeman, Z. (2022). Elderly care and burden of family carers during the first wave of COVID-19 pandemic. ORVOSI HETILAP, 163, 1654-1662. | No English translation |
| 14 | Maria, L.T.A., & Zsuzsa, S. (2022). Elderly care and burden of family carers during the first wave of COVID-19 pandemic. ORVOSI HETILAP, 163, 1654-1662. | No English translation |
| 15 | Czeisler, M.E., Lane, R.I., Petrosky, E., Wiley, J.F., Christensen, A., Njai, R., et al. (2020). Mental Health, Substance Use, and Suicidal Ideation During the COVID-19 Pandemic - United States, June 24-30, 2020. MMWR. Morbidity and mortality weekly report, 69, 1049-1057. | Not peer reviewed |
| 16 | Czeisler, M.E., Rohan, E.A., Melillo, S., Matjasko, J.L., DePadilla, L., Patel, C.G., et al. (2021). Mental Health Among Parents of Children Aged <18 Years and Unpaid Caregivers of Adults During the COVID-19 Pandemic - United States, December 2020 and February-March 2021. MMWR. Morbidity and mortality weekly report, 70, 879-887. | Not peer reviewed |
| 17 | Altieri, M., & Santangelo, G. (2021). The Psychological Impact of COVID-19 Pandemic and Lockdown on Caregivers of People With Dementia. The American journal of geriatric psychiatry : official journal of the American Association for Geriatric Psychiatry, 29, 27-34. | Wrong (or no) comparator |
| 18 | Anderson, S., Parmar, J., L'Heureux, T., Dobbs, B., Charles, L., & Tian, P.G.J. (2022). Family Caregiving during the COVID-19 Pandemic in Canada: A Mediation Analysis. International Journal of Environmental Research and Public Health, 19. | Wrong (or no) comparator |
| 19 | Chafouleas, S.M., & Iovino, E.A. (2021). Comparing the initial impact of COVID-19 on burden and psychological distress among family caregivers of children with and without developmental disabilities. School psychology (Washington, D.C.), 36, 358-366. | Wrong (or no) comparator |
| 20 | Ergenekon, A.P., Yilmaz Yegit, C., Cenk, M., Bas Ikizoglu, N., Atag, E., Gokdemir, Y., et al. (2021). Depression and anxiety in mothers of home ventilated children before and during COVID-19 pandemic. Pediatric pulmonology, 56, 264-270. | Wrong (or no) comparator |
| 21 | Fong, H.X., Cornish, K., Kirk, H., Ilias, K., Shaikh, M.F., & Golden, K.J. (2021). Impact of the COVID-19 Lockdown in Malaysia: An Examination of the Psychological Well-Being of Parent-Child Dyads and Child Behavior in Families With Children on the Autism Spectrum. Frontiers in psychiatry, 12, 733905. | Wrong (or no) comparator |
| 22 | Fusar-Poli, L., Martinez, M., Surace, T., Meo, V., Patania, F., Avanzato, C., et al. (2022). The Psychological Impact of the COVID-19 Lockdown: A Comparison between Caregivers of Autistic and Non-Autistic Individuals in Italy. Brain Sciences, 12. | Wrong (or no) comparator |
| 23 | Horiuchi, S., Shinohara, R., Otawa, S., Akiyama, Y., Ooka, T., Kojima, R., et al. (2020). Caregivers' mental distress and child health during the COVID-19 outbreak in Japan. PloS one, 15, e0243702. | Wrong (or no) comparator |
| 24 | Iannattone, S., Raffagnato, A., Zanato, S., Traverso, A., Tascini, B., Del Col, L., et al. (2021). Children with Psychopathology and Their Parents Facing the Covid-19 Pandemic: A Case-Control Study. Clinical neuropsychiatry, 18, 324-333. | Wrong (or no) comparator |
| 25 | Iovino, E.A., Caemmerer, J., & Chafouleas, S.M. (2021). Psychological distress and burden among family caregivers of children with and without developmental disabilities six months into the COVID-19 pandemic. Research in developmental disabilities, 114, 103983. | Wrong (or no) comparator |
| 26 | Joshi, D., Gonzalez, A., Griffith, L., Duncan, L., MacMillan, H., Kimber, M., et al. (2021). The trajectories of depressive symptoms among working adults during the COVID-19 pandemic: a longitudinal analysis of the InHamilton COVID-19 study. BMC Public Health, 21, 1895. | Wrong (or no) comparator |
| 27 | Kalb, L.G., Badillo-Goicoechea, E., Holingue, C., Riehm, K.E., Thrul, J., Stuart, E.A., et al. (2021). Psychological distress among caregivers raising a child with autism spectrum disorder during the COVID-19 pandemic. Autism research : official journal of the International Society for Autism Research, 14, 2183-2188. | Wrong (or no) comparator |
| 28 | Nakamoto, I., Murayama, H., Takase, M., Muto, Y., Saito, T., & Tabuchi, T. (2022). Association between increased caregiver burden and severe psychological distress for informal caregivers during the COVID-19 pandemic in Japan: A cross-sectional study. Archives of Gerontology and Geriatrics, 102, 104756. | Wrong (or no) comparator |
| 29 | Onay, Z.R., Gursoy, T.R., Eyuboglu, T.S., Aslan, A.T., Soysal Acar, A.S., Yapar, D., et al. (2021). Anxiety Levels of Primary Caregivers of Children With Tracheostomy During the COVID-19 Pandemic. Clinical pediatrics, 60, 504-511. | Wrong (or no) comparator |
| 30 | Polonyiova, K., Belica, I., Celusakova, H., Jansakova, K., Kopcikova, M., Szapuova, Z., et al. (2022). Comparing the impact of the first and second wave of COVID-19 lockdown on Slovak families with typically developing children and children with autism spectrum disorder. Autism, 26, 1046-1055. | Wrong (or no) comparator |
| 31 | Wei, Y., Li, Z., Guo, L., Zhang, L., Lian, C., Yuan, C., et al. (2023). The Moderating Role of Family Resilience on the Relationship between COVID-19-Related Psychological Distress and Mental Health among Caregivers of Individuals with Eating Disorders in Post-Pandemic China. International Journal of Environmental Research and Public Health, 20. | Wrong (or no) comparator |
| 32 | Willner, P., Rose, J., Stenfert Kroese, B., Murphy, G.H., Langdon, P.E., Clifford, C., et al. (2020). Effect of the COVID-19 pandemic on the mental health of carers of people with intellectual disabilities. Journal of applied research in intellectual disabilities : JARID, 33, 1523-1533. | Wrong (or no) comparator |
| 33 | Xue, B., & McMunn, A. (2021). Gender differences in unpaid care work and psychological distress in the UK Covid-19 lockdown. PloS one, 16, e0247959. | Wrong (or no) comparator |
| 34 | Aljuaid, M., Ilyas, N., Altuwaijri, E., Albedawi, H., Alanazi, O., Shahid, D., et al. (2022). Quality of Life among Caregivers of Patients Diagnosed with Major Chronic Disease during COVID-19 in Saudi Arabia. Healthcare (Basel, Switzerland), 10. | Wrong (or no) comparator |
| 35 | Angwenyi, V., Kabue, M., Chongwo, E., Mabrouk, A., Too, E.K., Odhiambo, R., et al. (2021). Mental Health during COVID-19 Pandemic among Caregivers of Young Children in Kenya's Urban Informal Settlements. A Cross-Sectional Telephone Survey. International Journal of Environmental Research and Public Health, 18. | Wrong (or no) comparator |
| 36 | Bloom, J., Lachman, A., Gaxo, E., Pillay, J., & Seedat, S. (2022). Child, adolescent, and caregiver mental health difficulties and associated risk factors early in the COVID-19 pandemic in South Africa. Child and adolescent psychiatry and mental health, 16, 65. | Wrong (or no) comparator |
| 37 | Deeb, S., Madden, D., Ghebretinsae, T., Lin, J., Ozbek, U., Mayer, V., et al. (2022). Child Disruptions, Remote Learning, and Parent Mental Health during the COVID-19 Pandemic. International Journal of Environmental Research and Public Health, 19. | Wrong (or no) comparator |
| 38 | Davidson, B., Schmidt, E., Mallar, C., Mahmoud, F., Rothenberg, W., Hernandez, J., et al. (2021). Risk and resilience of well-being in caregivers of young children in response to the COVID-19 pandemic. Translational behavioral medicine, 11, 305-313. | Wrong (or no) comparator |
| 39 | Di Gessa, G., Bordone, V., & Arpino, B. (2022). Changes in Grandparental Childcare During the Pandemic and Mental Health: Evidence From England. The journals of gerontology. Series B, Psychological sciences and social sciences. | Wrong (or no) comparator |
| 40 | Friesen, K.A., Weiss, J.A., Howe, S.J., Kerns, C.M., & McMorris, C.A. (2022). Mental Health and Resilient Coping in Caregivers of Autistic Individuals during the COVID-19 Pandemic: Findings from the Families Facing COVID Study. Journal of autism and developmental disorders, 52, 3027-3037. | Wrong (or no) comparator |
| 41 | Jafari-Oori, M., Ebadi, A., Moradian, S.T., Jafari, M., Dehi, M., & Ghasemi Fard, F. (2022). Psychiatric distress in family caregivers of patients with COVID-19. Archives of psychiatric nursing, 37, 69-75. | Wrong (or no) comparator |
| 42 | Kerr, M.L., Rasmussen, H.F., Fanning, K.A., & Braaten, S.M. (2021). Parenting During COVID-19: A Study of Parents' Experiences Across Gender and Income Levels. Family Relations, 70, 1327-1342. | Wrong (or no) comparator |
| 43 | Ferizi, M.M., Aali, S., Tavalaei, A.M., Bigdeli, I., & Rezaeitalab, F. (2022). COVID-19 Pandemic and Caregiving of Old Adults with Chronic Nervous System Disease. Iranian Journal of Psychiatry and Behavioral Sciences, 16, e122542. | Wrong (or no) comparator |
| 44 | Kimura, M., Kimura, K., & Ojima, T. (2021). Relationships between changes due to COVID-19 pandemic and the depressive and anxiety symptoms among mothers of infants and/or preschoolers: A prospective follow-up study from pre-COVID-19 Japan. BMJ Open, 11, e044826. | Wrong (or no) comparator |
| 45 | Lane, N.E., Hoben, M., Amuah, J.E., Hogan, D.B., Baumbusch, J., Gruneir, A., et al. (2022). Prevalence and correlates of anxiety and depression in caregivers to assisted living residents during COVID-19: a cross-sectional study. BMC Geriatrics, 22, 662. | Wrong (or no) comparator |
| 46 | Kurata, S., Hiraoka, D., Ahmad Adlan, A.S., Jayanath, S., Hamzah, N., Ahmad-Fauzi, A., et al. (2021). Influence of the COVID-19 Pandemic on Parenting Stress Across Asian Countries: A Cross-National Study. Frontiers in Psychology, 12, 782298. | Wrong (or no) comparator |
| 47 | L'Heureux, T., Parmar, J., Dobbs, B., Charles, L., Tian, P.G.J., Sacrey, L.-A., et al. (2022). Rural Family Caregiving: A Closer Look at the Impacts of Health, Care Work, Financial Distress, and Social Loneliness on Anxiety. Healthcare (Basel, Switzerland), 10. | Wrong (or no) comparator |
| 48 | Li, Q., Zhang, H., Zhang, M., Li, T., Ma, W., An, C., et al. (2021). Mental Health Multimorbidity among Caregivers of Older Adults During the COVID-19 Epidemic. The American journal of geriatric psychiatry : official journal of the American Association for Geriatric Psychiatry, 29, 687-697. | Wrong (or no) comparator |
| 49 | Kostyal, L.A., Szeman, Z., Almasi, V.E., Fabbietti, P., Quattrini, S., Socci, M., et al. (2022). The Impact of COVID-19 on the Health and Experience of the Carers of Family Members Living with Dementia: An Italian-Hungarian Comparative Study. International Journal of Environmental Research and Public Health, 19, 5329. | Wrong (or no) comparator |
| 50 | Price, A.M., Measey, M.-A., Hoq, M., Rhodes, A., & Goldfeld, S. (2022). Child and caregiver mental health during 12 months of the COVID-19 pandemic in Australia: findings from national repeated cross-sectional surveys. BMJ paediatrics open, 6. | Wrong (or no) comparator |
| 51 | Levante, A., Petrocchi, S., Bianco, F., Castelli, I., Colombi, C., Keller, R., et al. (2021). Psychological Impact of COVID-19 Outbreak on Families of Children with Autism Spectrum Disorder and Typically Developing Peers: An Online Survey. Brain Sciences, 11. | Wrong (or no) comparator |
| 52 | Li, Q., Zhang, H., Zhang, M., Li, T., Ma, W., An, C., et al. (2020). Prevalence and Risk Factors of Anxiety, Depression, and Sleep Problems Among Caregivers of People Living With Neurocognitive Disorders During the COVID-19 Pandemic. Frontiers in psychiatry, 11, 590343. | Wrong (or no) comparator |
| 53 | Ng, K.Y.Y., Zhou, S., Tan, S.H., Ishak, N.D.B., Goh, Z.Z.S., Chua, Z.Y., et al. (2020). Understanding the Psychological Impact of COVID-19 Pandemic on Patients With Cancer, Their Caregivers, and Health Care Workers in Singapore. JCO global oncology, 6, 1494-1509. | Wrong (or no) comparator |
| 54 | Price, A.M.H., Measey, M.A., Hoq, M., Rhodes, A., & Goldfeld, S. (2021). Child and caregiver mental health during 12 months of the COVID-19 pandemic in Australia: findings from national repeated cross-sectional surveys. medRxiv. | Wrong (or no) comparator |
| 55 | Russell, B.S., Hutchison, M., Tambling, R., Tomkunas, A.J., & Horton, A.L. (2020). Initial Challenges of Caregiving During COVID-19: Caregiver Burden, Mental Health, and the Parent-Child Relationship. Child psychiatry and human development, 51, 671-682. | Wrong (or no) comparator |
| 56 | Russell, B.S., Hutchison, M., Park, C.L., & Fendrich, M. (2021). SHORT-TERM IMPACTS OF COVID-19 ON FAMILY CAREGIVERS: STRESS APPRAISALS, COPING, AND MENTAL HEALTH. ANNALS OF BEHAVIORAL MEDICINE, 55, S414-S414. | Wrong (or no) comparator |
| 57 | Wade, M., Prime, H., Johnson, D., May, S.S., Jenkins, J.M., & Browne, D.T. (2021). The disparate impact of COVID-19 on the mental health of female and male caregivers. Social science & medicine (1982), 275, 113801. | Wrong (or no) comparator |
| 58 | Wang, C. (2021). Mental health and social support of caregivers of children and adolescents with ASD and other developmental disorders during COVID-19 pandemic. Journal of affective disorders reports, 6, 100242. | Wrong (or no) comparator |
| 59 | Wister, A., Li, L., Mitchell, B., Wolfson, C., McMillan, J., Griffith, L.E., et al. (2022). Levels of Depression and Anxiety Among Informal Caregivers During the COVID-19 Pandemic: A Study Based on the Canadian Longitudinal Study on Aging. The journals of gerontology. Series B, Psychological sciences and social sciences, 77, 1740-1757. | Wrong (or no) comparator |
| 60 | Yuan, S., Zhang, W., Yao, Q., Lu, W., Yu, W., Zhong, F., et al. (2022). The neuropsychiatric changes after COVID-19 quarantine in patients with cognitive impairment and their caregivers in Chongqing, China: A cohort study. Frontiers in Aging Neuroscience, 13. | Wrong (or no) comparator |
| 61 | Rajagopalan, J., Arshad, F., Thomas, P.T., Varghese, F., Hurzuk, S., Hoskeri, R.M., et al. (2022). Cognition, Behavior, and Caregiver Stress in Dementia during the COVID-19 Pandemic: An Indian Perspective. Dementia and geriatric cognitive disorders, 51, 90-100. | Wrong (or no) comparator |
| 62 | Xia, C.-L., Wei, A.-P., & Huang, Y.-T. (2022). The COVID-19 Lockdown and Mental Wellbeing of Females in China. International Journal of Environmental Research and Public Health, 19. | Wrong (or no) comparator |
| 63 | Xu, Y., Jedwab, M., Wu, Q., Levkoff, S.E., & Xu, L. (2022). Risk and protective factors associated with grandparent kinship caregivers' psychological distress in COVID-19: Kinship license status as a moderator. Child & family social work, 27, 41-54. | Wrong (or no) comparator |
| 64 | Zhang, L., Wu, M.T., Guo, L., Zhu, Z.Y., Peng, S.F., Li, W., et al. (2021). Psychological distress and associated factors of the primary caregivers of offspring with eating disorder during the coronavirus disease 2019 pandemic. Journal of Eating Disorders, 9, 58. | Wrong (or no) comparator |
| 65 | Zhang, X. (2022). Household Chaos and Caregivers' and Young Children's Mental Health during the COVID-19 Pandemic: A Mediation Model. Journal of child and family studies, 31, 1547-1557. | Wrong (or no) comparator |
| 66 | Wauters, A., Vervoort, T., Dhondt, K., Soenens, B., Vansteenkiste, M., Morbee, S., et al. (2022). Mental health outcomes among parents of children with a chronic disease during the COVID-19 pandemic: The role of parental burn-out. Journal of pediatric psychology, 47, 420-431. | Wrong (or no) comparator |
| 67 | Tambling, R., Russell, B., Tomkunas, A., Horton, A., & Hutchison, M. (2021). Factors Contributing to Parents' Psychological and Medical Help Seeking During the COVID-19 Global Pandemic. Family & community health, 44, 87-98. | Wrong (or no) comparator |
| 68 | Elugbadebo, O.O., & Baiyewu, O. (2022). Mild anxiety and depression disorders: Unusual reactions to COVID-19 lockdown in caregivers of older adults attending a psychogeriatric clinic in Southwest Nigeria. The Nigerian postgraduate medical journal, 29, 13-19. | Wrong (or no) comparator |
| 69 | Abdeta, T., & Desalegn, D. (2021). Common Mental Disorders Among Informal Primary Caregivers of Adults With Mental Illness During the Coronavirus Disease 2019 Epidemic in Eastern Ethiopia: A Cross-Sectional Study. Frontiers in psychiatry, 12, 676379. | Wrong (or no) comparator |
| 70 | Akdag, B., Onder, A., Gizli Coban, O., Kocacik Uygun, D.F., Surer Adanir, A., Erdem, A., et al. (2022). Psychological State of Parents of Children with Primary Immunodeficiencies During the COVID-19 Pandemic. Pediatric allergy, immunology, and pulmonology, 35, 12-18. | Wrong (or no) comparator |
| 71 | Alessi, J., de Oliveira, G.B., Feiden, G., Schaan, B.D., & Telo, G.H. (2021). Caring for caregivers: the impact of the COVID-19 pandemic on those responsible for children and adolescents with type 1 diabetes. Scientific Reports, 11, 6812. | Wrong (or no) comparator |
| 72 | Carpinelli Mazzi, M., Iavarone, A., Musella, C., De Luca, M., de Vita, D., Branciforte, S., et al. (2020). Time of isolation, education and gender influence the psychological outcome during COVID-19 lockdown in caregivers of patients with dementia. European Geriatric Medicine, 11, 1095-1098. | Wrong (or no) comparator |
| 73 | Czeisler, M.E., Drane, A., Winnay, S.S., Capodilupo, E.R., Czeisler, C.A., Rajaratnam, S.M., et al. (2021). Mental health, substance use, and suicidal ideation among unpaid caregivers of adults in the United States during the COVID-19 pandemic: Relationships to age, race/ethnicity, employment, and caregiver intensity. Journal Of Affective Disorders, 295, 1259-1268. | Wrong (or no) comparator |
| 74 | Czeisler, M.E., Wiley, J.F., Facer-Childs, E.R., Robbins, R., Weaver, M.D., Barger, L.K., et al. (2021). Mental health, substance use, and suicidal ideation during a prolonged COVID-19-related lockdown in a region with low SARS-CoV-2 prevalence. Journal of Psychiatric Research, 140, 533-544. | Wrong (or no) comparator |
| 75 | Maytles, R., Bergman, Y.S., & Trachtingot, I. (2021). Caregiving burden and depressive symptoms among ultra-orthodox jews: The moderating role of sense of community. Journal of community & applied social psychology, No-Specified. | Wrong (or no) comparator |
| 76 | Messina, A., Lattanzi, M., Albanese, E., & Fiordelli, M. (2022). Caregivers of people with dementia and mental health during COVID-19: findings from a cross-sectional study. BMC Geriatrics, 22, 56. | Wrong (or no) comparator |
| 77 | Nwosu, C.O. (2021). Childcare and depression during the coronavirus pandemic in South Africa: A gendered analysis. PloS one, 16, e0255183. | Wrong (or no) comparator |
| 78 | Todorovic, N., Vracevic, M., Rajovic, N., Pavlovic, V., Madzarevic, P., Cumic, J., et al. (2020). Quality of Life of Informal Caregivers behind the Scene of the COVID-19 Epidemic in Serbia. Medicina (Kaunas, Lithuania), 56. | Wrong (or no) comparator |
| 79 | Zwar, L., Konig, H.-H., & Hajek, A. (2022). Gender Differences in Mental Health, Quality of Life, and Caregiver Burden among Informal Caregivers during the Second Wave of the COVID-19 Pandemic in Germany: A Representative, Population-Based Study. Gerontology, 1-14. | Wrong (or no) comparator |
| 80 | Yuan, S., Zhang, W., Lu, W., Yu, W., Zhong, F., Xiong, L., et al. (2021). The psychological impact on patients with memory disorders and their caregivers during COVID-19. Aging clinical and experimental research, 33, 2317-2325. | Wrong (or no) comparator |
| 81 | Radomski, A., Cloutier, P., Polihronis, C., Gardner, W., Pajer, K., Sheridan, N., et al. (2022). Parenting during the COVID-19 pandemic: The sociodemographic and mental health factors associated with maternal caregiver strain. Families, systems & health : the journal of collaborative family healthcare, 40, 79-86. | Wrong (or no) comparator |
| 82 | Urizar, G.G., Jr., Ramirez, I., Caicedo, B.I., & Mora, C. (2022). Mental health outcomes and experiences of family caregivers of children with disabilities during the COVID-19 pandemic in Bolivia. Journal of Community Psychology, 50, 2682-2702. | Wrong (or no) comparator |
| 83 | Rajovic, T., Todorovic, N., Vracevic, M., Rajovic, N., Pavlovic, A., Pavlovic, V., et al. (2021). From Burden to Depressive Symptoms in Informal Caregivers during the COVID-19 Pandemic: A Path Analysis. International Journal of Environmental Research and Public Health, 18. | Wrong (or no) comparator |
| 84 | Aryee, E., Perrin, J.M., Clancy, S., Merrill, C., Curran, M., & Oreskovic, N.M. (2023). Mental Health of Caregivers of Children with Medical Complexity During COVID-19. Journal of developmental and behavioral pediatrics : JDBP. | Wrong (or no) comparator |
| 85 | Engelbrecht, M. (2023). Factors Associated with COVID-19-Related Stress among Female Primary Caregivers in Vulnerable Families in South Africa. European journal of investigation in health, psychology and education, 13, 377-390. | Wrong (or no) comparator |
| 86 | Wei, G., Diehl-Schmid, J., Matias-Guiu, J.A., Pijnenburg, Y., Landin-Romero, R., Bogaardt, H., et al. (2022). The effects of the COVID-19 pandemic on neuropsychiatric symptoms in dementia and carer mental health: an international multicentre study. Scientific Reports, 12. | Wrong (or no) comparator |
| 87 | Zwar, L., K..nig, H.-H., & Hajek, A. (2023). Gender Differences in Mental Health, Quality of Life, and Caregiver Burden among Informal Caregivers during the Second Wave of the COVID-19 Pandemic in Germany: A Representative, Population-Based Study. Gerontology, 69, 149-162. | Wrong (or no) comparator |
| 88 | Newby, J.M., O'Moore, K., Tang, S., Christensen, H., & Faasse, K. (2020). Acute mental health responses during the COVID-19 pandemic in Australia. PloS one, 15, e0236562. | Wrong (or no) comparator |
| 89 | Anderson, S., Parmar, J., Dobbs, B., & Tian, P.G.J. (2021). A Tale of Two Solitudes: Loneliness and Anxiety of Family Caregivers Caring in Community Homes and Congregate Care. International Journal of Environmental Research and Public Health, 18. | Wrong (or no) comparator |
| 90 | Farajzadeh, A., Dehghanizadeh, M., Maroufizadeh, S., Amini, M., & Shamili, A. (2021). Predictors of mental health among parents of children with cerebral palsy during the COVID-19 pandemic in Iran: A web-based cross-sectional study. Research in developmental disabilities, 112, 103890. | Wrong (or no) comparator |
| 91 | Dominke, C., Wei, G., Piguet, O., Kumfor, F., & Diehl-Schmid, J. (2021). Impact of the COVID-19 pandemic on neuropsychiatric symptoms of patients with dementia and the mental health of their caregivers. Nervenheilkunde, 40, 861-869. | Wrong (or no) comparator |
| 92 | Bao, X., Xu, J., Meng, Q., Gan, J., Wang, X.-D., Wu, H., et al. (2022). Impact of the COVID-19 Pandemic and Lockdown on Anxiety, Depression and Nursing Burden of Caregivers in Alzheimer's Disease, Dementia With Lewy Bodies and Mild Cognitive Impairment in China: A 1-Year Follow-Up Study. Frontiers in psychiatry, 13, 921535. | Wrong (or no) comparator |
| 93 | Franza, F., Basta, R., Solomita, B., Conte, G., Vacca, A., Chung, S., et al. (2021). Anxiety and burnout in alzheimer's caregivers in COVID-19 pandemic. Journal of the neurological sciences, 429, 119961. | Wrong (or no) comparator |
| 94 | Leggett, A., Koo, H.J., Park, B., & Choi, H. (2022). The Changing Tides of Caregiving During the COVID-19 Pandemic: How Decreasing and Increasing Care Provision Relates to Caregiver Well-Being. The journals of gerontology. Series B, Psychological sciences and social sciences, 77, S86-S97. | Wrong (or no) comparator |
| 95 | Alexopoulos, P., Soldatos, R., Kontogianni, E., Frouda, M., Loanna Aligianni, S., Skondra, M., et al. (2021). COVID-19 Crisis Effects on Caregiver Distress in Neurocognitive Disorder. Journal of Alzheimer's disease : JAD, 79, 459-466. | Wrong exposure |
| 96 | Di Sarno, E., & Louza, M.R. (2023). The burden of caregivers of schizophrenia outpatients during the COVID-19 pandemic: A same-sample comparison with the pre-pandemic burden. The International journal of social psychiatry, 207640231156512. | Wrong exposure |
| 97 | Ehrlich, U., Kelle, N., Klaus, D., & Mohring, K. How did the COVID-19 pandemic impact the wellbeing of family care-givers? A longitudinal study of older adults in Germany. Ageing & Society. | Wrong exposure |
| 98 | Price, A.M.H., Contreras-Suarez, D., Zhu, A., Schreurs, N., Measey, M.A., Woolfenden, S., et al. (2021). The relationships between ongoing COVID-19 lockdown and the financial and mental health experiences of Australian families. medRxiv. | Wrong exposure |
| 99 | Banerjee, S., Mukherjee, A., Bhattacharyya, B., Mohanakumar, K.P., & Biswas, A. (2022). Quality of life and Concerns of Parkinson's Disease Patients and their Caregivers during COVID-19 Pandemic: An Indian Study. Annals of Indian Academy of Neurology, 25, 676-682. | Wrong exposure |
| 100 | Connor, C., De Valliere, N., Warwick, J., Stewart-Brown, S., & Thompson, A. (2022). The COV-ED Survey: exploring the impact of learning and teaching from home on parent/carers' and teachers' mental health and wellbeing during COVID-19 lockdown. BMC Public Health, 22, 889. | Wrong exposure |
| 101 | Gamble, L.D., Parker, S., Quinn, C., Bennett, H.Q., Martyr, A., Sabatini, S., et al. (2022). A Comparison of Well-Being of Carers of People with Dementia and Their Ability to Manage Before and During the COVID-19 Pandemic: Findings from the IDEAL Study. Journal of Alzheimer's disease : JAD, 88, 679-692. | Wrong exposure |
| 102 | Garg, R.K., Garg, K., Chopra, V., Gupta, N., & Bharti, R. (2022). Psychosocial Impact of Pandemic and State Imposed Lockdown on Caregivers of Patients Presenting with Respiratory Complaints Mimicking COVID-19: A Short-term Follow-up Study. Journal of Clinical and Diagnostic Research, 16, LC34-LC39. | Wrong exposure |
| 103 | Geweniger, A., Barth, M., Haddad, A.D., Hogl, H., Insan, S., Mund, A., et al. (2022). Impact of the COVID-19 Pandemic on Mental Health Outcomes of Healthy Children, Children With Special Health Care Needs and Their Caregivers-Results of a Cross-Sectional Study. Frontiers in pediatrics, 10, 759066. | Wrong exposure |
| 104 | Dhiman, S., Sahu, P.K., Reed, W.R., Ganesh, G.S., Goyal, R.K., & Jain, S. (2020). Impact of COVID-19 outbreak on mental health and perceived strain among caregivers tending children with special needs. Research in developmental disabilities, 107, 103790. | Wrong exposure |
| 105 | Miniarikova, E., Vernhet, C., Peries, M., Loubersac, J., Picot, M.-C., Munir, K., et al. (2022). Anxiety and depression in parents of children with autism spectrum disorder during the first COVID-19 lockdown: Report from the ELENA cohort. Journal of Psychiatric Research, 149, 344-351. | Wrong exposure |
| 106 | Morelen, D., Najm, J., Wolff, M., & Daniel, K. (2022). Taking care of the caregivers: The moderating role of reflective supervision in the relationship between COVID-19 stress and the mental and professional well-being of the IECMH workforce. Infant mental health journal, 43, 55-68. | Wrong exposure |
| 107 | Miller, K.E.M., Van Houtven, C.H., Smith, V.A., Lindquist, J.H., Gray, K., Richardson, C., et al. (2022). Family Caregivers of Veterans Experience Clinically Significant Levels of Distress Prepandemic and During Pandemic: Implications for Caregiver Support Services. Medical Care, 60, 530-537. | Wrong exposure |
| 108 | Van Gorp, M., Maurice-Stam, H., Teunissen, L., Van De Peppel-Van Der Meer, W., Huussen, M., Schouten-Van Meeteren, A., et al. (2021). No Increase in Psychosocial Stress of Dutch Children with Cancer and Their Caregivers during the Covid-19 Pandemic. Pediatric Blood and Cancer, 68. | Wrong exposure |
| 109 | van Gorp, M., Maurice-Stam, H., Teunissen, L.C., Kilsdonk, E., van Dijk, J., Sulkers, M., et al. (2022). Psychosocial function of Dutch children with cancer and their caregivers during different phases of the COVID-19 pandemic. Pediatric blood & cancer, 69, e29535. | Wrong exposure |
| 110 | Whiting, D., Atee, M., Morris, T., & Cunningham, C. (2021). Effect of COVID-19 on BPSD severity and caregiver distress: Trend data from national dementia-specific behavior support programs in Australia. Alzheimer's & dementia : the journal of the Alzheimer's Association, 17, e058454. | Wrong exposure |
| 111 | Mohanty, J., Chokkanathan, S., & Alberton, A.M. (2022). COVID-19-related stressors, family functioning and mental health in Canada: Test of indirect effects. Family Relations, 71, 445-462. | Wrong exposure |
| 112 | Alegria, M., Cruz-Gonzalez, M., O'Malley, I.S., Alvarez, K., Stein, G.L., Fuentes, L., et al. (2022). Role of social determinants in anxiety and depression symptoms during COVID-19: A longitudinal study of adults in North Carolina and Massachusetts. Behaviour research and therapy, 154, 104102. | Wrong exposure |
| 113 | Calear, A.L., McCallum, S., Morse, A.R., Banfield, M., Gulliver, A., Cherbuin, N., et al. (2022). Psychosocial impacts of home-schooling on parents and caregivers during the COVID-19 pandemic. BMC Public Health, 22, 119. | Wrong exposure |
| 114 | Gordon, J.L., & Presseau, J. (2022). Effects of parenthood and gender on well-being and work productivity among Canadian academic research faculty amidst the COVID-19 pandemic. Canadian Psychology / Psychologie canadienne, No-Specified. | Wrong exposure |
| 115 | Nikolaidou, E., Tsatali, M., Eleftheriou, M., Wang, H., Karagiozi, K., Margaritidou, P., et al. (2022). Emotional Function, Negative Thoughts about the Pandemic, and Adaptability Skills among Dementia Caregivers during the COVID-19 Pandemic. Brain Sciences, 12. | Wrong exposure |
| 116 | Li, J.H., Bunning, M., Kaiser, T., & Hipp, L. (2022). Who suffered most? Parental stress and mental health during the COVID-19 pandemic in Germany. JFR-JOURNAL OF FAMILY RESEARCH, 34, 280-306. | Wrong exposure |
| 117 | Baiden, P., LaBrenz, C.A., & Findley, E. (2021). Social distancing and anxiety among female caregivers of children ages zero-to-five during coronavirus disease (COVID-19) lockdown in the United States. Journal of affective disorders reports, 5, 100154. | Wrong exposure |
| 118 | Brown, E.M., Fernald, L.C.H., Hamad, R., Hoskote, M., Jackson, K.E., & Gosliner, W. (2022). Pandemic-related socioeconomic disruptions and adverse health outcomes: a cross-sectional study of female caregivers. BMC Public Health, 22, 1893. | Wrong exposure |
| 119 | Nakanishi, M., Richards, M., Stanyon, D., Yamasaki, S., Endo, K., Sakai, M., et al. (2022). Adolescent Carers' Psychological Symptoms and Mental Well-being During the COVID-19 Pandemic: Longitudinal Study Using Data From the UK Millennium Cohort Study. The Journal of adolescent health : official publication of the Society for Adolescent Medicine, 70, 877-884. | Exposure (caregiving) not measured during the pandemic |
| 120 | Wister, A., Li, L., Levasseur, M., Kadowaki, L., & Pickering, J. (2022). The Effects of Loneliness on Depressive Symptoms Among Older Adults During COVID-19: Longitudinal Analyses of the Canadian Longitudinal Study on Aging. Journal of Aging and Health, 8982643221129686. | Exposure (caregiving) not measured during the pandemic |
| 121 | Otobe, Y., Kimura, Y., Suzuki, M., Koyama, S., Kojima, I., & Yamada, M. (2022). Factors Associated with Increased Caregiver Burden of Informal Caregivers during the COVID-19 Pandemic in Japan. The journal of nutrition, health & aging, 26, 157-160. | Wrong outcome |
| 122 | Taniguchi, Y., Miyawaki, A., Tsugawa, Y., Murayama, H., Tamiya, N., & Tabuchi, T. (2022). Family caregiving and changes in mental health status in Japan during the COVID-19 pandemic. Archives of Gerontology and Geriatrics, 98, 104531. | Wrong outcome |
| 123 | Graler, L., Bremmers, L., Bakx, P., van Exel, J., & van Bochove, M. (2022). Informal care in times of a public health crisis: Objective burden, subjective burden and quality of life of caregivers in the Netherlands during the COVID-19 pandemic. Health & Social Care in the Community. | Wrong outcome |
| 124 | Park, C., & Kim, M. (2022). Stressors associated with older adults' depressive symptom during the pandemic: Does a caregiving role make a difference? Social Work in Mental Health, No-Specified. | Wrong outcome |
| 125 | Truskinovsky, Y., Finlay, J.M., & Kobayashi, L.C. (2022). Caregiving in a Pandemic: COVID-19 and the Well-Being of Family Caregivers 55+ in the United States. Medical care research and review : MCRR, 79, 663-675. | Wrong outcome |
| 126 | Bergmann, M., & Wagner, M. (2021). The Impact of COVID-19 on Informal Caregiving and Care Receiving Across Europe During the First Phase of the Pandemic. Frontiers in public health, 9, 673874. | Wrong outcome |
| 127 | Carballo, J.L., Coloma-Carmona, A., Arteseros-Banon, S., & Perez-Jover, V. (2021). The Moderating Role of Caregiving on Fear of COVID-19 and Post-Traumatic Stress Symptoms. International Journal of Environmental Research and Public Health, 18. | Wrong outcome |
| 128 | Biliunaite, I., Kazlauskas, E., Sanderman, R., & Andersson, G. (2022). Informal caregiver support needs and burden: a survey in Lithuania. BMJ Open, 12, e054607. | Wrong outcome |
| 129 | Maggio, M.G., La Rosa, G., Calatozzo, P., Andaloro, A., Foti Cuzzola, M., Cannavo, A., et al. (2021). How COVID-19 Has Affected Caregivers' Burden of Patients with Dementia: An Exploratory Study Focusing on Coping Strategies and Quality of Life during the Lockdown. Journal of clinical medicine, 10. | Wrong outcome |
| 130 | Pena-Longobardo, L.M., Oliva-Moreno, J., & Rodriguez-Sanchez, B. (2022). The Effects of Severe Acute Respiratory Syndrome Coronavirus 2 on the Reported Mental Health Symptoms of Nonprofessional Carers: An Analysis Across Europe. Value in health : the journal of the International Society for Pharmacoeconomics and Outcomes Research, 25, 736-743. | Wrong outcome |
| 131 | Busse, C., Barnini, T., Zucca, M., Rainero, I., Mozzetta, S., Zangrossi, A., et al. (2022). Depression, Anxiety and Sleep Alterations in Caregivers of Persons With Dementia After 1-Year of COVID-19 Pandemic. Frontiers in psychiatry, 13, 826371. | No effect estimate/s for the association in question |
| 132 | Landi, G., Pakenham, K.I., Cattivelli, R., Grandi, S., & Tossani, E. (2022). Caregiving Responsibilities and Mental Health Outcomes in Young Adult Carers during the COVID-19 Pandemic: A Longitudinal Study. International Journal of Environmental Research and Public Health, 19. | No effect estimate/s for the association in question |
| 133 | Gallagher, S., & Wetherell, M.A. (2020). Risk of depression in family caregivers: unintended consequence of COVID-19. BJPsych open, 6, e119. | Same sample as Whitley 2021 |
| 134 | Seck, P.A., Encarnacion, J.O., Tinonin, C., & Duerto-Valero, S. (2021). Gendered Impacts of COVID-19 in Asia and the Pacific: Early Evidence on Deepening Socioeconomic Inequalities in Paid and Unpaid Work. Feminist Economics, 27, 117-132. | Wrong study design |
| 135 | Chan, E.Y.Y., Lo, E.S.K., Huang, Z., Kim, J.H., Hung, H., Hung, K.K.C., et al. (2020). Characteristics and well-being of urban informal home care providers during COVID-19 pandemic: A population-based study. BMJ Open, 10, 041191. | Wrong study design |
| 136 | Cohen, S.A., Nash, C.C., & Greaney, M.L. (2021). Informal Caregiving During the COVID-19 Pandemic in the US: Background, Challenges, and Opportunities. AMERICAN JOURNAL OF HEALTH PROMOTION, 35, 1032-1036. | Wrong study design |
| 137 | Adesina, M.A., Olufadewa, I.I., Oladele, R.I., Abudu, F.R., Lawal, T., & Ayelawa, S.D. (2022). Depression and anxiety during the COVID-19 pandemic. Psychiatria, 19, 171-175. | Wrong study design |
